# Epigenetic regulation of *TP53* is involved in prostate cancer radioresistance and DNA damage response signaling

**DOI:** 10.1038/s41392-023-01639-6

**Published:** 2023-10-16

**Authors:** Catarina Macedo-Silva, Vera Miranda-Gonçalves, Nuno Tiago Tavares, Daniela Barros-Silva, Joana Lencart, João Lobo, Ângelo Oliveira, Margareta P. Correia, Lucia Altucci, Carmen Jerónimo

**Affiliations:** 1 Cancer Biology & Epigenetics Group, Research Center of IPO Porto (CI-IPOP)/ CI-IPOP@ RISE (Health Research Network), Portuguese Oncology Institute of Porto (IPO-Porto)/Porto Comprehensive Cancer Center Raquel Seruca (Porto.CCC), R. Dr. António Bernardino de Almeida, 4200-072 Porto, Portugal; 2 Department of Precision Medicine, University of Campania “Luigi Vanvitelli”, 80138 Naples, Italy; 3 Department of Pathology and Molecular Immunology, ICBAS-School of Medicine & Biomedical Sciences, University of Porto, R. Jorge de Viterbo Ferreira 228, 4050-313 Porto, Portugal; 4 Medical Physics, Radiobiology and Radiation Protection Group—Research Center of IPO Porto (CI-IPOP)/CI-IPOP@ RISE (Health Research Network), Portuguese Oncology Institute of Porto (IPO-Porto)/Porto Comprehensive Cancer Center Raquel Seruca (Porto.CCC), R. Dr. António Bernardino de Almeida, 4200-072 Porto, Portugal; 5 Department of Medical Physics, Portuguese Oncology Institute of Porto, 4200-072 Porto, Portugal; 6 Department of Pathology, Portuguese Oncology Institute of Porto, Porto, Portugal; 7 Department of Radiation Oncology, Portuguese Oncology Institute of Porto, Porto, Portugal; 8 BIOGEM, Molecular Biology and Genetics Research Institute, 83100 Avellino, Italy; 9 IEOS, Institute of Endocrinology and Oncology, 80100 Naples, Italy

**Keywords:** Cancer therapy, Urological cancer

## Abstract

External beam radiotherapy (RT) is a leading first-line therapy for prostate cancer (PCa), and, in recent years, significant advances have been accomplished. However, RT resistance can arise and result in long-term recurrence or disease progression in the worst-case scenario. Thus, making crucial the discovery of new targets for PCa radiosensitization. Herein, we generated a radioresistant PCa cell line, and found p53 to be highly expressed in radioresistant PCa cells, as well as in PCa patients with recurrent/disease progression submitted to RT. Mechanism dissection revealed that RT could promote p53 expression via epigenetic modulation. Specifically, a decrease of H3K27me3 occupancy at *TP53* gene promoter, due to increased KDM6B activity, was observed in radioresistant PCa cells. Furthermore, p53 is essential for efficient DNA damage signaling response and cell recovery upon stress induction by prolonged fractionated irradiation. Remarkably, KDM6B inhibition by GSK-J4 significantly decreased p53 expression, consequently attenuating the radioresistant phenotype of PCa cells and hampering in vivo 3D tumor formation. Overall, this work contributes to improve the understanding of p53 as a mediator of signaling transduction in DNA damage repair, as well as the impact of epigenetic targeting for PCa radiosensitization.

## Introduction

Under stressful circumstances, cell survival depends on the ability to properly activate DNA damage signaling responses.^1^ Radiotherapy (RT) is one of the main genotoxic cell-killing therapies for a wide range of tumors.^1^ Ionizing radiation (IR)-induced DNA damage commonly triggers the activation of several cell-programmed responses, including early transduction pathways of DNA sensing effectors to double-strand break (DSB) repair, as well as cell cycle arrest or apoptosis.^1,2^ Frequently, DNA replication stress in aggressive cancers generates genomic instability, driving tumor cell-killing escape through phosphorylation of γ-H2AX followed by the activation of DNA damage repair (DDR) early players, such as ATM/ATR, BRCA1, the trimeric protein complex MRN, CHK2, and XLF, which are detected at DSB foci.^1–4^ Targeting the deregulation of such players has been suggested as a potential strategy for radiosensitization. The success of cancer therapies, such as RT, relies on the knowledge of the molecular basis and proliferative behavior of each tumor model and their cell division properties.^5^ Radiosensitizers targeting DNA damage responses are promising strategies to overcome therapy ineffectiveness, preventing tumor regrowth.^4^

Efforts to counteract the setback of tumor cell radioresistance continue to be carried out, although little progress has been accomplished for prostate cancer (PCa), which remains a highly prevalent and aggressive disease among men, worldwide.^6^ Ionizing radiation-based therapies represent an important and effective first-line approach for PCa.^6,7^ Unfortunately, intermediate to high-risk PCa patients are commonly prone to develop long-term recurrence or experience short-term disease progression upon RT due to the emergence of resistance.^7^ Specifically, prostate adenocarcinomas are considered late RT-responding tumors, disclosing high ability to repair sublethal damage in a short time period after RT, exceptionally presenting relatively low α/β ratios comparing to other tumor models.^8^ Overall, relatively higher doses are required for effective cell injury in PCa.^8^ Thus, moderate hypofractionated regimens are often prescribed for PCa patients, using daily delivered doses of 2.5 Gy for a total dose of 70 Gy.^8,9^ Despite marked advances in this field, high rates of therapeutic failure remain.^7^

The p53 status is related with key functions in PCa radiosensitivity.^10^ However, its intrinsic cellular regulation upon radiation exposure is not yet fully understood in this context. The dual function of p53 may result in either adaptive survival or induction of cell death.^10^ Proteasomal degradation of p53 is avoided when damage repair mechanisms need to be activated.^11^ Hence, p53 plays a key role in maintaining the integrity of the genome. Indeed, p53 deficient cells are prone to lose the sparing effect to small RT fractions.^12^ Remarkably, histone H3 post-translational modifications such as methylation, have been reported as main drivers of *TP53* transcriptional preservation.^13,14^ Specifically, histone-associated repressive marks are key modifiers responsible for the structural dynamics of chromatin, particularly at the *TP53* gene promoter.^13^

Overall, in concomitance with current standard therapies, novel targetable molecules which may allow for tumor downstaging, are urgently needed. Hence, epigenetic targeting of *TP53* might constitute a valid strategy for PCa radiosensitization. Thus, we sought to identify clinically relevant DNA damage signaling proteins and determine whether its epigenetic regulation might impact in PCa patients’ clinical outcomes. We found that p53 and KDM6B were overexpressed in PCa cells exposed to RT and that both contributed to PCa radioresistance.

## Results

### *TP53* overexpression and proficient DNA damage repair in PCa radioresistant cells

**Fig. 1:**
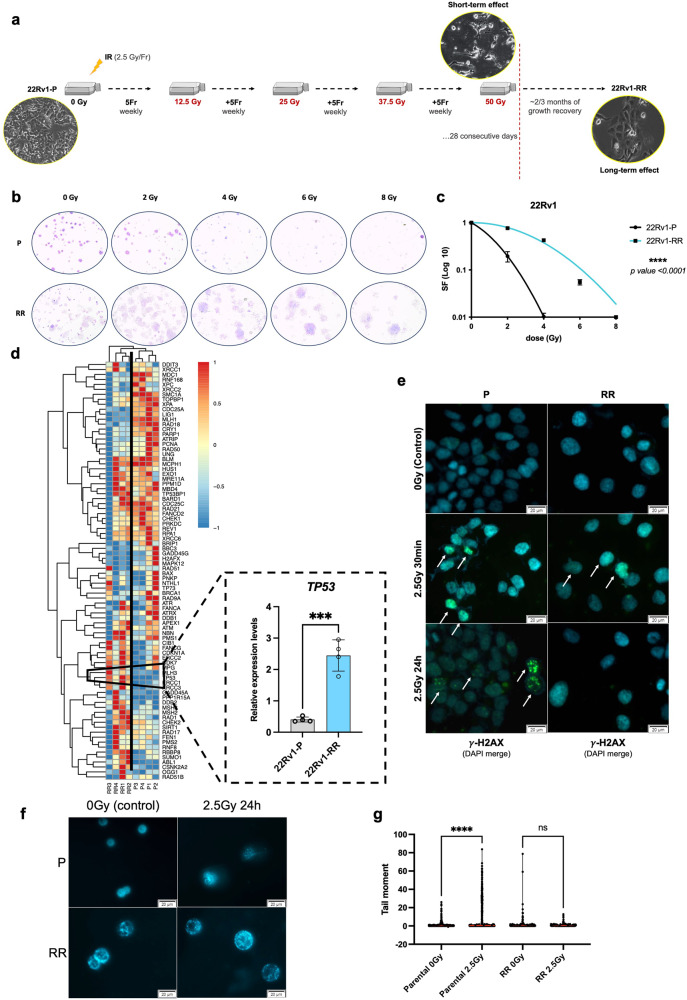
DNA damage signaling in radioresistant 22Rv1 (RR) PCa cell lines. **a** Schematic representation of 22Rv1-RR in vitro generation upon 20 fractions of 2.5 Gy for 28 consecutive days for a total dose of 50 Gy. Cells recover the growth properties within ~2–3 months in culture after the acute effect of ionizing radiation. Morphological differences were observed in cells between short-term and long-term effect of ionization. **b** Representative images of the stained colonies submitted to a range of 0 to 8 Gy. Images were taken by stereomicroscope Olympus S2X16 using a digital camera Olympus SC180, 0.7×. **c** Cell survival fraction of 22Rv1-RR and parental (P) cells represented through linear-quadratic model (LQ = (S = e ^– (xD + BD2)^)), ****P value < 0.0001. **d** Heatmap of RT^2^ Profiler Human DNA Damage Signaling Pathway PCR array comparing between 22Rv1-P and 22Rv1-RR samples (four biological replicates), highlighting relative mRNA expression levels of *TP53*, normalized by *HPRT1* gene expression levels (housekeeping). In blue, less expressed samples (−1) and in red, high expressed samples (1). Results are presented as mean ± SD of at least three independent experiments. ***P value < 0.001. **e** Immunofluorescence staining of γ-H2AX foci (merged with DAPI for nuclei staining) in 22Rv1-P and RR cells control (0 Gy) and after 2.5 Gy IR (30 min and 24 h after). Images were taken using Olympus IX51 microscope at ×400 magnification (scale bar 20 μm). **f**, **g** Effect of 2.5 Gy irradiation treatment in DNA damage of 22Rv1-P and RR cells by comet assay. The results are the mean of at least 100 comets per condition. ns not significant; **P value < 0.01; ***P value < 0.001; ****P value < 0.0001. Representative images of the DAPI-stained single cells were taken using Olympus IX51 microscope at 400x magnification (scale bar 20 μm). The graph represents tail moment which means tail length × % of DNA in the tail. Red line represents the median value. Fr fraction, IR ionizing radiation, P parental, RR radioresistant

Here, we established a PCa radioresistant cell line model via exposing 22Rv1 parental (P) cells to increasing weekly doses of multifractionated (MF) irradiation. Briefly, we induced radioresistance in 22Rv1-P cells upon a cumulative dose of up to 50 Gy by using multifractionated (MF) irradiation (2.5 Gy/fraction; 5 fractions/week; 4 weeks), as schematically represented in Fig. 1a. Significant increase in clonogenicity was confirmed for 22Rv1-RR cells (Fig. 1b). When submitted to a range of 0–8 Gy, in single-dose (SD), the survival fraction of 22Rv1-RR cells was significantly higher compared to the parental cell line (Fig. 1c).

To disclose differentially expressed genes with important functions in DNA damage repair possibly involved in induced radioresistance, we performed a DNA damage signaling qPCR array for 22Rv1-P and 22Rv1-RR. Remarkably, a statistically significant upregulation of *TP53* was observed in 22Rv1-RR cells (Fig. 1d). Indeed, *TP53* displayed almost six-fold higher mRNA levels, with a *P* value lower than 0.001 (Supplementary Table S1). Nonetheless, in addition to p53, global deregulation of DNA damage repair (DDR)-related genes was observed in our RR cell model, as well (Fig. 1d and Supplementary Table S1). Thus, our results support the role of p53 as a DDR initiator. Remarkably, instantaneous phosphorylation of the core histone variant H2AX at serine 136 is commonly detected at DSB sites, acting as a DNA damage sensor.^15^ We found that although some immunofluorescent foci appeared after 30 min of IR, complete γ-H2AX resolution was observed in 22Rv1-RR cells after 24 h of 2.5 Gy in SD-IR (Fig. 1e). On the other hand, delayed or imperceptible resolution of γ-H2AX was observed for 22Rv1-P cells (Fig. 1e). Likewise, increased percentage of DNA fragmentation (tail moment) was detected in 22Rv1-P cells after 24 h of SD-IR, suggesting greater propensity to withhold damage and/or lower ability to repair it in parental cells compared to 22Rv1-RR cells (Fig. 1f, g).

### Histone chromatin dynamics modulate *TP53* expression in PCa cells

**Fig. 2:**
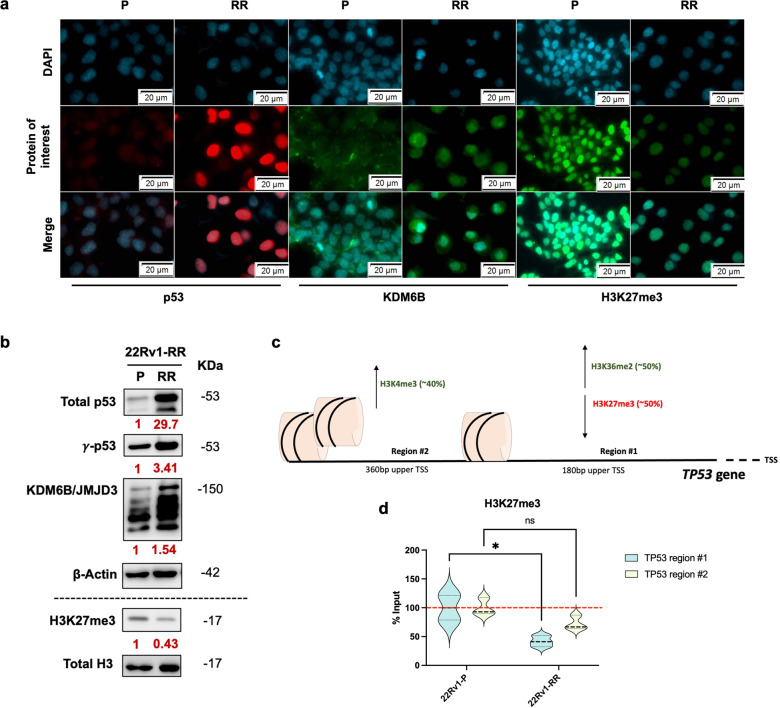
Epigenetic regulation of *TP53* gene promoter. **a** Nuclear immunofluorescent staining of p53 (red) and H3K27me3 (green) and cytoplasm/nuclear staining of KDM6B (green), for 22Rv1-P and 22Rv1-RR cells. DAPI images represent the nucleus location. Merge images represent the merge of DAPI and images of protein of interest. Images were taken using Olympus IX51 microscope at 400x magnification (scale bar 20 μm). **b** Total protein levels of p53 (53 kDa), γ-p53 (53 kDa), and KDM6B/JMJD3 (150 kDa) for 22Rv1-P and RR cells. β-actin (42 kDa) was used as loading control (upper). Histone protein levels of H3K27me3 (17 kDa) for 22Rv1-P and RR cells. Total H3 (17 kDa) was used as a loading control (down). The values below each target represent the optical density average of the fold change between 22Rv1-RR and 22Rv1-P, measured using ImageJ tools. The images were taken by Chemidoc detection system (Biorad, Berkeley, California). Optical density values were obtained using ImageJ software version 1.53 (National Institutes of Health). Values are representative of RR vs. P of at least three independent replicates. **c** Representative scheme of *TP53* gene promoter under epigenetic regulation. Transcription activation-related markers are marked in green and transcription repression-related markers in red. **d** The graphs represent H3K27me3 %input values (ChIP-qPCR) at TP53 gene promoter in regions #1 and #2 (180 bp and 360 bp upper TSS, respectively). The violin plots depict mean ± SD of at least three independent replicates. ns, non-significant; *P value < 0.05; ***P value < 0.001; ****P value < 0.0001. bp base pairs, P parental, RR radioresistant, TSS transcription starting site

In line with the transcriptionally overexpressed *TP53*, higher p53 protein levels were confirmed in 22Rv1-RR cells (Fig. 2a, b). Furthermore, phosphorylation of p53 (γ-p53) at serine 15 occurs upon DSB formation, and it is a key negative regulator of p53 degradation, preserving its functions as DNA damage repair mediator.^16^ Indeed, after prolonged exposure to fractionation IR, higher γ-p53 protein levels were observed (Fig. 2b). Those results were also observed in an additional PCa cell line, C4-2B, exposed to prolonged fractionated ionization, with significantly higher clonogenicity compared with the parental cell lines (Supplementary Fig. S1). In another aggressive cell line, DU145, p53 expression was already high under baseline conditions (Supplementary Fig. S1).

Previous reports suggested that *TP53* gene promoter might be regulated through epigenetic modifications, including histone methylation dynamics.^17,18^ Thus, we hypothesized whether epigenetic modulation of *TP53* expression could be associated with PCa radioresistance after the course of prolonged fractionated irradiation. Indeed, in addition to higher nuclear p53 protein expression (Fig. 2a, b), a KDM6B cytoplasm-nucleus shift and overall increased protein expression was observed in 22Rv1-RR cells compared to parental cells (Fig. 2a, b). Conversely, decreased nuclear H3K27me3 levels, a well-known repressive histone mark and KDM6B target, was found in RR cells (Fig. 2a, b). Altogether, these results suggest higher KDM6B/JMJD3 activity induced by fractionation IR-induced radioresistance.

To evaluate whether KDM6B epigenetically interacts with *TP53* promoter and exerts its demethylase activity upon H3K27, we performed chromatin immunoprecipitation experiments. Notably, KDM6B immunoprecipitation was higher at *TP53* gene promoter in 22Rv1-RR cells than in the parental cells (Supplementary Fig. S2a, b), whereas H3K27me3 was reduced in 22Rv1-RR cells (Fig. 2c, d). Furthermore, KDM6B only co-precipitated with p53 in 22Rv1-RR cells (Supplementary Fig. S2c) and a slight increase in relative H3K4me3 and H3K36me2 (both transcription activation marks) deposit was observed at the *TP53* promoter (Supplementary Fig. S2d, e). No significant differences were found for H3K9me2 and EZH2 binding (Supplementary Fig. S2f, g).

Overall, these results indicate *TP53* gene promoter transcriptional activation in 22Rv1-RR cells, compared with parental cells, due to a general decrease in histone repressors and a parallel increase in activators.

### P53 upregulation in PCa patients with recurrent/progressive disease and its impact on patient survival

**Fig. 3:**
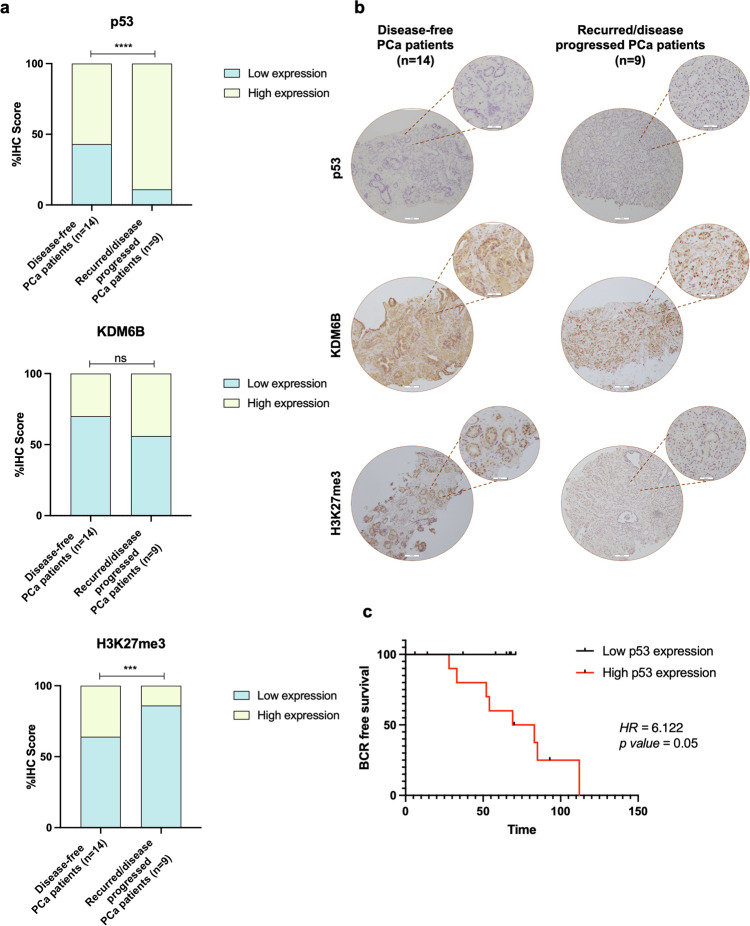
p53, KDM6B, and H3K27me3 immunoexpression in a series of PCa tissues from patients who experience or not recurrences after RT treatment. **a** p53, KDM6B, and H3K27me3 IHC score values comparing recurrent/progressive disease with disease-free PCa patients, represented by contingency graphs. Fisher’s exact test was used to determine statistically significant differences between the two groups. ns non-significant; ***P value < 0.001; ****P value < 0.0001. **b** Representative IHC images for p53, KDM6B, and H3K27me3 protein expression in PCa tissues for both groups of disease-free and recurrent/progressive PCa patients. Images were taken using Olympus BX41 microscope with a digital camera Olympus U-TV0.63XC in CellSens software (version V0116, Olympus), at ×100 magnification and ×200 (larger and smaller circle, scale bar 100 μm and 200 μm, respectively). **c** Biochemical recurrence (BCR) free survival analysis in months discriminated according to low or high p53 expression levels. Hazard ratio (HR) risk of 6.122 and *P* value = 0.05

Considering that p53 status may have implications for the outcome of patients exposed to radiation therapy,^19^ we assessed its expression in PCa patients submitted to RT. We found that p53 immunoexpression was significantly higher in pre-treatment PCa patients who experienced biochemical recurrence (BCR) or disease progression after RT treatment (Fig. 3a, b). Specifically, 8 (~89%) out of 9 PCa patients with disease progression/recurrence after RT disclosed high p53 immunoexpression score (Fig. 3a). Accordingly, although not statistically significant, higher KDM6B nuclear levels were observed for the same patients (Fig. 3a, b). Nonetheless, significantly lower levels of its specific histone target, H3K27me3, were observed in samples from the group with the worst prognosis (Fig. 3a, b). Taken together, these results are in line with the in vitro findings on PCa 22Rv1-RR cells.

Moreover, patients displaying higher p53 expression experienced worse prognosis, displaying six times more chance of biochemical recurrence than patients with lower p53 levels at diagnosis (Fig. 3c). However, no associations were found between KDM6B or H3K27me3 expression and patients’ outcome (data not shown).

### Epigenetic mitigation of p53 expression by GSK-J4 attenuates PCa radioresistance

**Fig. 4:**
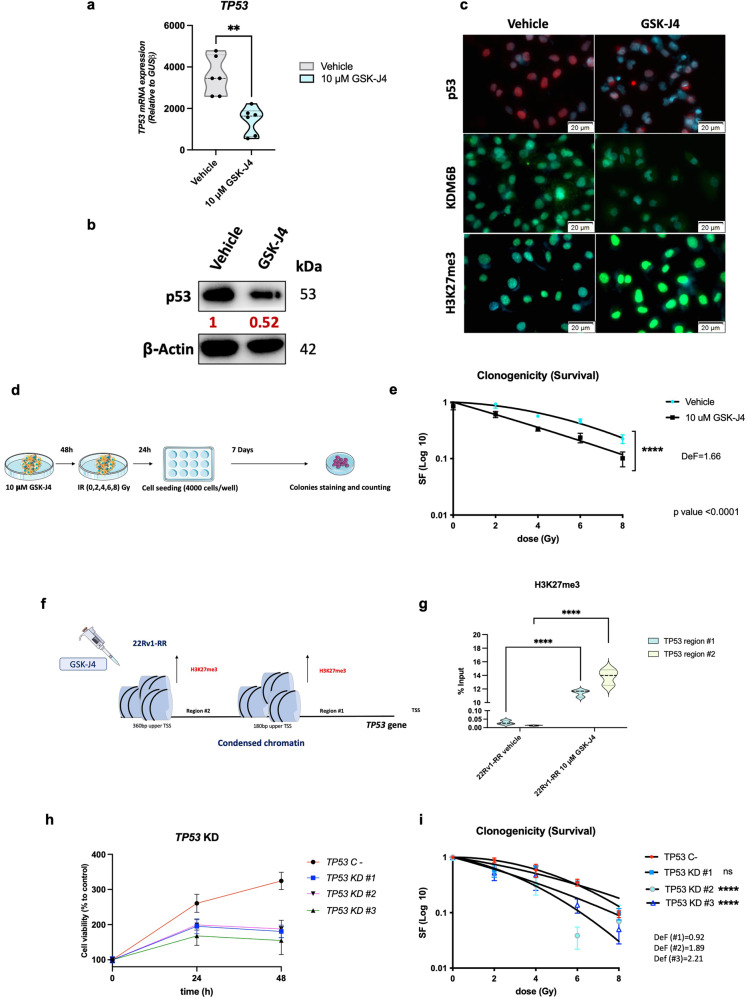
*TP53* epigenetic silencing leads to the mitigation of radioresistant phenotype in 22Rv1-RR cells. **a** Relative mRNA expression levels of *TP53* gene for 22Rv1-RR GSK-J4-treated cells and vehicle control (DMSO), normalized by *GUSB* gene expression levels (housekeeping). Results are presented as mean ± SD of at least three independent experiments. **P value < 0.01. **b** Total protein levels of p53 (53 kDa) to compare between 10 μM GSK-J4-treated 22Rv1-RR cells and the vehicle (DMSO). β-actin (42 kDa) was used as loading control. Images were taken by the Chemidoc detection system (Biorad, Berkeley, California). Optical density values were obtained using ImageJ software version 1.53 (National Institutes of Health). Values are representative of GSK-J4 vs. vehicle of at least three independent replicates. **c** DAPI merged images of nuclear immunofluorescent staining of p53 (red) and H3K27me3 (green) and cytoplasm/nuclear staining of KDM6B (green), for 22Rv1-RR GSK-J4-treated cells and vehicle control (DMSO). Images were taken using Olympus IX51 microscope at ×400 magnification (scale bar 20 μm). **d** Schematic representation of colony formation assay experiments for GSK-J4 treatment. **e** Cell survival fraction of 22Rv1-RR treated with 10 μM of GSK-J4 and vehicle cells represented through linear-quadratic model (LQ = (S = e ^– (xD + BD2)))^, ****P value < 0.0001, with a dose-enhancement factor (DeF) of 1.66. **f** Representative scheme of TP53 promoter epigenetic regulation upon GSK-J4 treatment in 22Rv1-RR cells. In red is represented the transcription repression-related marker, H3K27me3. **g** The graph represents H3K27me3 %input values (ChIP-qPCR) at *TP53* gene promoter in regions #1 and #2 (180 bp and 360 bp upper TSS, respectively). The violin plots were represented by mean ± SD of at least three independent replicates. ns non-significant; **P value < 0.01; ****P value < 0.0001. **h** Cell viability assay for 22Rv1-RR negative control (TP53 C-) and TP53-KD clones #1 to #3 at 24 h and 48 h. **i** Cell survival fraction of 22Rv1-RR-negative control (TP53 C-) and TP53-KD clones #1 to #3 transfected cells represented through linear-quadratic model (LQ = (S = e – (αD + βD2))), ns, non-significant; ****P value < 0.0001. Results are presented as mean ± SD of at least 3 independent experiments. bp base pairs, Def, dose enhancement factor, KD knockdown, RR radioresistant, TSS transcription starting site

Because our results indicated that p53 expression was partially regulated by histone methylation dynamics at its gene promoter, we further addressed whether KDM6B targeting by GSK-J4, a potent inhibitor of H3K27me3-demethylases, could influence PCa radiosensitivity via indirect p53 modulation. Remarkably, we found that GSK-J4 treatment, as well as *KDM6B* knockdown (KD) in 22Rv1-RR cells, significantly flattened p53 levels, leading to significant sensitization to IR exposure (Fig. 4a–e and Supplementary Fig. S3). Besides the decrease of nuclear p53 in 22Rv1-RR cells, treatment with 10 μM of GSK-J4 led to superficial changes in KDM6B protein levels and a substantial increase of H3K27me3 in the same cell line, suggesting less enzymatic activity (Fig. 4c).

Notably, the clonogenicity of 22Rv1-RR cells significantly decreased with 10 μM of GSK-J4 treatment (Fig. 4d, e). Thus, inhibition of KDM6B attenuated the radioresistant phenotype of 22Rv1-RR cells (Fig. 4d, e). The radiosensitizing effect of GSK-J4 was represented by a dose-enhancement factor (DeF) of 1.66 (ID_50_ vehicle/ID_50_ drug), where ID_50_ corresponds to the required dose for 50% growth inhibition^20^ (Fig. 4e).

Furthermore, 22Rv1-RR cells exposed to 10 μM of GSK-J4 treatment disclosed a significant increase of H3K27me3 occupancy at *TP53* promoter region (Fig. 4f, g). Together, these results support the hypothesis that *TP53* transcription is regulated by epigenetic modulation.

Next, we ascertained whether *TP53* knockdown induced the same effects in cell viability as GSK-J4 treatment, to discard the hypothesis of toxicity effects and to prove that radioresistant properties of 22Rv1-RR cells are indeed p53 dependent. We, thus, performed CRISPR/Cas9 knockdown using three different knocked-down clones. We confirmed that *TP53* gene was knocked down compared to the negative control, both at transcript and protein levels (Supplementary Fig. S4). Notably, *TP53* knockdown (KD) phenocopied the radiosensitizing effect of GSK-J4, highlighting the importance of p53 in radiation response for PCa (Fig. 4h, i). Specifically, *TP53*-*KD* 22Rv1-RR cells disclosed significantly reduced cell viability compared to the negative control (Fig. 4h). Moreover, *TP53*-KD clones #2 and #3 depicted significantly reduced clonogenicity upon exposure to IR-SD of 0–8 Gy (Fig. 4i), with DeF values of 1.89 and 2.21, respectively.

### GSK-J4 treatment impaired DNA damage recovery in 22Rv1-RR cells

**Fig. 5:**
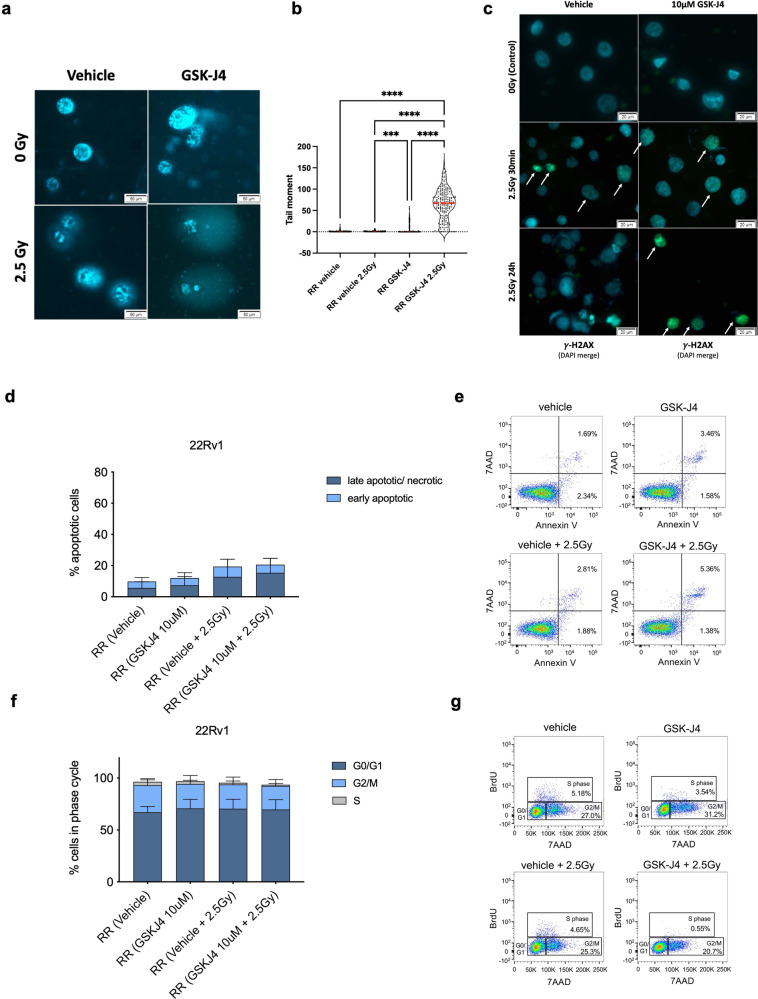
Impaired DNA damage repair, proliferation, and increased apoptosis in 22Rv1-RR GSK-J4-treated cells. **a**, **b** Effect of 2.5 Gy irradiation treatment in DNA damage of 22Rv1-RR GSK-J4-treated cells and vehicle control (DMSO) by comet assay. The results are the mean of at least 100 comets per condition. *P value < 0.05; ***P value < 0.001; ****P value < 0.0001. The graph represents tail moment, corresponding to tail length × % of DNA in the tail. Red line represents the median value. **c** Immunofluorescence staining of γ-H2AX foci (merged with DAPI) in 22Rv1-RR treated with GSK-J4 and vehicle control (DMSO) at 0 Gy and after 2.5 Gy IR (30 min and 24 h after). Images were taken using Olympus IX51 microscope at ×400 magnification (scale bar 20 μm). **d**, **e** Data quantification by flow cytometry for early and late apoptotic % cells and representative Annexin V/7ADD staining dot plots. **f**, **g** Representative graphs and dot plots for cell cycle analysis by flow cytometry according to BrdU/7ADD cell staining

To better understand the effect of combined treatment with GSK-J4 and IR in 22Rv1-RR cells, functional assays related to cellular stress/DNA damage and cell division were performed. The combo-treatment significantly increased DNA damage compared with IR, GSK-J4 or the vehicle control group alone (Fig. 5a, b). Indeed, increased DNA fragmentation (tail moment) was observed with 10 μM of GSK-J4, being more evident upon combo-treatment (GSK-J4 + 2.5 Gy) (Fig. 5b). In both vehicle and GSK-J4 treatment, γ-H2AX foci were detected after 30 min of 2.5 Gy exposure (Fig. 5c). However, only GSK-J4-treated cells maintained high γ-H2AX levels after 24 h of IR exposure, whereas the vehicle group recovered from the damage (Fig. 5c).

We further addressed the impact of GSK-J4 treatment on cell death and cell cycle redistribution. Increased percentage of late apoptosis/necrosis was observed both upon combo-treatment (10 μM of GSK-J4 + 2.5 Gy IR) and IR alone, comparing with vehicle control cells, while only marginal differences were observed upon GSK-J4 treatment alone (Fig. 5d, e). As expected, 22Rv1-RR displayed lower percentage of apoptotic cells than parental 22Rv1 cells (Supplementary Fig. S5a). Interestingly, upon IR exposure in combination with GSK-J4 treatment, 22Rv1-RR apoptotic levels increased to levels similar to parental 22Rv1 cells (Supplementary Fig. S5b). Although minor changes were found in cell cycle redistribution, a considerable decrease in S and G2/M proliferative phases was observed in combo-treatment, compared with the vehicle (Fig. 5f, g and Supplementary Fig. S5c, d).

Taken together, these results indicate that GSK-J4 significantly impairs 22Rv1-RR cell’s DNA damage recovery without a major impact on cell cycle distribution or apoptosis.

### GSK-J4 attenuates PCa radioresistance: impairment of 3D tumor formation in vivo

**Fig. 6:**
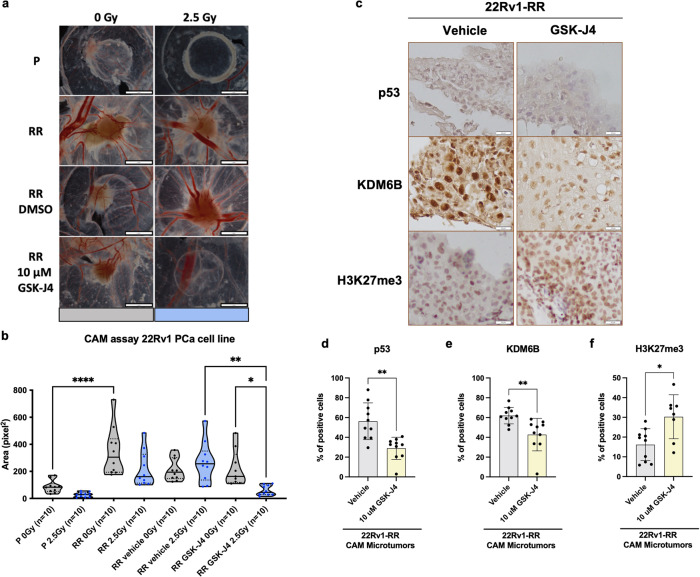
GSK-J4 impaired in vivo microtumor formation of 22Rv1-RR cells. **a** Representative images of CAM microtumors for 22Rv1-P, RR, RR vehicle (DMSO), and RR GSK-J4-treated cells with or without expsure to 2.5 Gy of IR-SD. Digital images were taken under a stereomicroscope Olympus S2X16 using a digital camera Olympus SC180. **b** Relative area (pixel^2^) of the formed microtumors was assessed using ImageJ software tools. Results are presented as mean ± SD of ten eggs per group condition; *P < 0.05; **P < 0.01; ****P < 0.001. **c** Representative IHC images for p53, KDM6B, and H3K27me3 protein expression in CAM-assay microtumors tissue sections for both groups of vehicle and GSK-J4. Pictures were taken using Olympus BX41 microscope with a digital camera Olympus U-TV0.63XC in CellSens software (version V0116, Olympus), at ×400 magnification (scale bar, 20 μm). **d** p53, **e** KDM6B, and **f** H3K27me3—% of positive staining cells in vehicle and GSK-J4 treatment groups. Scatter plot bar graphs depict mean ± SD of all selected microtumors. *P value < 0.05. CAM Chicken choriallantoic membrane, P parental, PCa prostate cancer, RR radioresistant, SD single-dose

Finally, we investigated whether GSK-J4 exposure might impair in vivo 3D tumor formation using chicken chorioallantoic membrane (CAM) as in vivo model system. Firstly, we observed a higher ability for 3D tumor formation in 22Rv1-RR cells compared to parental cells (Fig. 6a, b), with significant differences in area measurements (pixel^2^) (*P* value < 0.0001). Notably, GSK-J4 10 μM and 2.5 Gy IR combination significantly reduced 3D tumor-forming ability and microtumor area comparatively to drug or irradiation/vehicle alone (Fig. 6a, b). Furthermore, GSK-J4 treatment significantly reduced p53 and KDM6B immunoexpression, while enhancing H3K27me3 levels, closely mirroring in vitro results (Fig. 6c, d). Altogether, these results indicate that GSK-J4 attenuates the malignant phenotype of radioresistant PCa cells in vivo.

### General discussion and conclusions

Presently, radiotherapy (RT) constitutes one of the standards of care for PCa. It is commonly prescribed with neoadjuvant hormone therapy for intermediate/high-risk PCa patients as a tumor downstaging strategy.^21^ The therapeutic efficacy has been improving over the years due to the implementation of new techniques such as image-guided and intensity-modulated RT (IGRT and IMRT).^21,22^ However, the unpredictability of RT resistance acquisition entails the need to develop new therapeutic strategies for those patients in which RT proves to be less effective.^23,24^ The main target of IR is the nuclear DNA molecule.^23^ The most lethal damage is the double-strand DNA break, which triggers immediate cellular responses that may either lead to cell repair or its demise.^23^

Indeed, upon IR-induced DNA damage, p53, the “guardian of the genome”, may either lead to recovery of the injured cell, through cell cycle control, or to its eradication, through apoptotic and/or senescence mechanisms.^25,26^ The dual function of this transcription factor, having key roles in cell fate decisions, makes its understanding even more challenging. *TP53* overexpression was observed in our in vitro generated radioresistant cells (22Rv1-RR), upon prolonged exposure to fractionated IR. Moreover, we were able to confirm p53 upregulation in diagnostic prostate biopsies of patients submitted to RT that recurred/progressed, compared to those that did not. Indeed, *TP53* is known to be frequently altered in cancer, including in PCa. It has been shown that decreased p53, by siRNA transfection, in DU145 PCa cells led to impaired homologous recombination (HR)-mediated DNA damage repair, improving RT responsiveness.^27^ Also, functional p53 restoration in p53-null PC3 cells resulted in increased clonogenicity upon radiation exposure.^10,28^ In accordance with our data, multiplex protein expression profiling of generated 22Rv1-RR cells revealed that 43 out of the 90 signaling proteins analyzed disclosed significant alterations comparatively to parental cells.^29^ Among them, higher p53 protein levels, as well as activation of Notch signaling were observed in RR cells.^29^ Altogether, these results emphasize the role of p53 as a driver of radioresistance in PCa. Interestingly, Stattin et al. reported, in 1996, that p53 immunoreactivity did not significantly correlate with PCa patient disease-specific survival. However, the cohort analyzed comprised only 60 PCa patients^30^ and these were not grouped according to the outcome of RT (recurrence/ no recurrence).^30^ Conversely, in 2002, Ritter et al. demonstrated that higher p53 indexes in pre-treatment biopsies significantly correlated with higher rates of PSA (biochemical) failure.^31^ Likewise, in our cohort, patients with higher p53 immuno-score displayed shorter recurrence-free survival in response to RT. Nonetheless, our series is small (*n* = 23), and the data must be interpreted with caution.

Although, over the years, several studies have indicated p53 as a transcription factor closely related to RT response, little is known about its intrinsic regulatory mechanisms.^1^ Furthermore, reliable and effective new radiosensitizing strategies based on p53 inactivation are scarce. KDM6B/JMJD3 catalyzes H3K27 demethylation and triggers the activation of several gene promoters. Specifically, increase of genome-wide binding of KDM6B and p53 was reported in human fibroblasts after IR.^18^ KDM6B was found transcriptionally activated in response to diverse stimuli, such as stress signal of IR-induced damage.^18^ Herein, we disclosed KDM6B upregulation in 22Rv1-RR cells. Both p53 and KDM6B proteins were upregulated and recruited in response to IR-induced DNA injury. Higher KDM6B enzymatic activity often results in lower H3K27me3 levels. Indeed, significantly reduced H3K27me3 was observed in 22rv1-RR cells, comparing with parental ones, as well as in recurrent/ progressive PCa patients submitted to RT. Interestingly, p53 and KDM6B were previously shown to be co-localized at several p53 genome binding sites, leading to the activation of genes specifically involved in DNA damage repair, cell cycle control, apoptosis and p53 stabilization.^18^ Furthermore, epigenetic regulation of *TP53* transcription might be related with post-translational histone modifications.^17,18^ Remarkably, we found that immunoprecipitation of KDM6B was higher, whereas that of its target, H3K27me3, was lower at *TP53* gene promoter in 22Rv1-RR cells, compared with the parental ones. Furthermore, KDM6B only co-precipitated with p53 in 22Rv1-RR cells, strengthening the hypothesis that these two proteins are co-players in PCa cells’ response to IR-induced injury, which is in line with published data.^18^ Investigation of such epigenetic alterations holds the promise of identifying clinically helpful targetable molecules to improve RT response rates of PCa patients.

Thus, based on our findings, we propose a model of radioresistant PCa in which KDM6B/p53 interactions lead to increased *TP53* transcriptional activity and subsequent p53 activation via phosphorylation, allowing for more efficient DNA damage repair, reducing cell death and delaying cell proliferation in response to IR. Indeed, under stress conditions, p53 mediates G2/M cell cycle transition arrest, preventing cells from entering mitosis.^32^ Hence, mitotic cells are hypersensitive to IR-induced damage. Based on this, our results suggest that p53 overexpressing cells have advantage over radiation-responsive cells. Interestingly, Jumanji C domain (JmjC) demethylase inhibitors have emerged as promising radiosensitizing strategies for different tumor models. Recently, we have shown the potential of IOX1, a pan-JmjC KDM inhibitor, as radiosensitizer for esophageal carcinoma under hypoxic conditions.^33^ Furthermore, targeting H3K4me3 and/or H3K9me3 with JIB-04 increased radiation sensitivity of non-small cell lung cancer, reducing the efficacy of DDR.^34^ Although these inhibitors demonstrated promising results in in vitro studies, their non-selective inhibitory activity is a major drawback. On the other hand, GSK-J4, which is highly specific for H3K27 demethylation, appears to have impact in diffuse intrinsic pontine glioma (DIPG) radiosensitivity, reducing the expression of IR-induced HR DNA DSB repair-related genes, such as *PCNA*, *XRCC1*, *FANCA*, and *POLD1*.^35^ In addition, apoptotic triggering was performed using simultaneous GSK-J4 and APR-246 (apoptosis inducer), targeting mutant p53 in the same tumor model.^36^ Remarkably, we found that treatment of 22Rv1-RR cells with GSK-J4 restored low p53 levels, consequently leading to significant sensitization to IR exposure. Likewise, we showed that *TP53* knockdown cells phenocopy the radiosensitizing effect of GSK-J4, highlighting the importance of p53 in the radiation response of PCa. Overall, using GSK-J4 as a radiosensitizing strategy we observed increased DSB foci formation through γ-H2AX detection, potentiating DNA fragmentation and, consequently, cell death. Hence, GSK-J4 appears to be a suitable complementary strategy for PCa patients who respond unfavorably to RT. In the same line, CAM 22Rv1-RR microtumors treated with the combination of 10 μM GSK-J4 and 2.5 Gy IR displayed significantly reduced tumor area. To the best of our knowledge, there are no clinical trials in progress testing JmjC inhibitors combined with RT. Indeed, only one phase I clinical trial for PCa patients, initiated in 2008, evaluated the combination of an epigenetic drug (LBH589, Panobinostat) with external beam RT for the treatment of prostate, esophageal and head and neck cancers (NCT00670553). However, dose-limiting toxicity results were not yet posted concerning this study.

Further investigation is needed in this field to overcome the setback of tumors which are less responsive to cell-killing therapies, such as RT. In this regard, our study emphasizes the importance of epigenetic targeting, highlighting the role of KDM6B-p53 interactions and its impact on DNA damage repair for the acquisition of RT resistance in PCa.

## Materials and methods

### Ethics statement for patient’s samples

This study used PCa biopsy specimens as FFPE tissue samples. For that purpose, this study was approved by the institutional review board (Comissão de Ética para a Saúde) of IPO Porto, Portugal (CES-238/020). All procedures involving the use of human samples were in accordance with the institutional/national ethical standards and following the 1964 Helsinki declaration and its later amendments or comparable ethical standards.

### Prostate cancer patient’s cohort

PCa tissue biopsies were carefully selected in a retrospective cohort of PCa patients which underwent RT treatment between 2011 and 2014 at Portuguese Oncology Institute of Porto (IPO Porto), Portugal. The series comprises diagnostic PCa biopsies with two different clinical outcomes for radiation therapy: disease-free (*n* = 14) and recurrent/progressive disease (*n* = 9) PCa. Androgen deprivation therapy or other type of therapies were not previously employed in any of these cases. The total group of 23 PCa patients of this cohort disclosed a biochemical recurrence rate of ~22% at 5 years of follow-up. Representative formalin-fixed paraffin-embedded (FFPE) tissue blocks were chosen considering the presence of tumor cells. All slides were histologically examined and confirmed after hematoxylin & eosin staining by an experienced Uropathologist.

### Cell culture and treatments

Human PCa cell lines, 22Rv1, C4-2B, and DU145, were cultured in RPMI 1640 (Biotecnómica, Porto, Portugal) supplemented with 10% of fetal bovine serum (FBS), 100 IU/mL penicillin and 100 µg/mL streptomycin. Cells were maintained at 37 °C in a humidifier incubator with 5% CO_2_ and 74% N_2_. Cells were periodically tested for Mycoplasma contamination using MyTaq HS Red PCR Mycoplasma Detection Set (Bioline, Meridian Bioscience, London, UK). Cell line authentication was performed by Genomics Scientific Platform at i3S – Institute for Research & Innovation in Health, University of Porto, Portugal. The complete identity of this cell line was confirmed by genotyping.

Cell permeable pro-drug GSK-J4 (Sigma Aldrich, Germany) is a histone lysine demethylase (KDM) inhibitor that prevents JMJD3/UTX-induced H3K27 methylation removal. For this study, GSK-J4 was used at a 10 μM concentration for several functional assays in 22Rv1-RR cell lines. Dimethyl sulfoxide (DMSO) was used as a vehicle.

### Ionizing radiation

All PCa cell lines were irradiated for in vitro assays, as previously described^33^. Radioresistant (RR) cell subpopulations were generated from their respective parental lineage (P) using a RT fractionation scheme following EAU guidelines for PCa treatment. Specifically, 22Rv1 human PCa cells were exposed to daily irradiation of 2.5 Gy in 20 fractions for a total dose of 50 Gy, for 28 consecutive days. This newly generated lineage was used as a RR model for further analysis and/or experimental assays.

### Clonogenicity assay

Single cells were plated in 6-well plates prior to IR exposure and/or GSK-J4 treatment and/or *TP53* silencing, at an optimized density and maintained in culture for 7 days after ionization radiation exposure in the range of 0 to 8 Gy. Afterward when colonies were formed, cells were stained using diffQuick cell staining reagents. Colonies were defined as a bulk of at least 50 cells.

### Generation of stable *TP53* knockdown cell lines using CRISPR/Cas9 technology

22Rv1-RR cells were used for *TP53* knockdown (KD) using lentivirus-based CRISPR/Cas9 system constructed with guide RNA sequences specifically targeting TP53 exon 1 genomic region. Two different sgRNAs were independently used for TP53 KD—oligo 1: CACCGATCTGACTGCGGCTCCTCCA, and oligo 2: AAACTGGAGGAGCCGCAGTCAGATC. Briefly, for plasmid transfection, Lipofectamine® 3000 reagent (Invitrogen, USA) was used, according to the manufacturer’s instructions. Then, efficiently transfected cells were selected using puromycin. Upon selection, cells were expanded for further protein extraction and WB validation. Next, clonal selection was performed using the serial dilution method in 96-well plates. Finally, three independent KD clones were used for phenotypic assays.

### Genomic DNA sequencing to confirm TP53 deletion by CRISPR/Cas9

Cells were collected, and genomic DNA (gDNA) was isolated using the phenol-chloroform method. Briefly, target regions amplification was performed with 400 ng of gDNA by conventional PCR using MyTaq HS Red polymerase enzyme and the following primers—forward (Fw) sequence: ATCCCCACTTTTCCTCTTGCAG; Reverse (Rv) sequence: GCCCTTCCAATGGATCCACTCA—at annealing temperature of 58 °C for 35 cycles. Then, the resulting PCR product was confirmed by Gel electrophoresis at 1% agarose. Supplementary Fig. S6 shows that sample #2, #3 and #4, representing *TP53*-KD clones #1 to #3, have heterozygous deletion of TP53. Sample #1 is the negative control (wild-type 22Rv1-RR cells) with complete WT sequence of *TP53*. Of note, 22Rv1 wild-type cells display a heterozygous missense mutation of p53 at position 992 (c.992 A > G)^37^. Furthermore, karyotype analysis of this cell line demonstrated a tetraploid genome (4n)^38^. PCR products for the deleted region and corresponding control were extracted from the agarose gel and purified using Qiaquick gel extraction kit #28706 (Qiagen, Germany), according to the manufacturer’s instructions. A second PCR was performed using BigDye Terminator reagent v3.1 Cycle Sequencing kit (Applied Biosystems™/Thermo Scientific Inc., USA) with either TP53 Fw or Rv primers and 1μL of PCR product/ExoSAP-it mixture. The last step was the purification of the final PCR product in Sephadex 50 (GE Healthcare, United States) resin. PCR products were used for Sanger sequencing in a 3500 Genetic Analyzer (Applied Biosystems™/Thermo Scientific Inc., USA). Sequencing data was analyzed using the Web-based tool (https://www.ebi.ac.uk/Tools/msa/clustalo/) and alignment sequences for *TP53* WT (negative control, C-), as well as *TP53*-KD clones #1 to #3 are shown in Supplementary Fig. S6.

### Protein extraction and SDS-PAGE western blot

Total protein extraction was performed as previously described^33^ using 22Rv1-P and -RR cell line extracts under different conditions: with GSK-J4 treatment and after TP53 gene silencing. Additionally, histone extracts were obtained using a lysis solution of 0.5% TritonX-100 in 1× phosphate-buffered saline (PBS 1x) after rotating twice for 10 min. Then, after cell centrifugation at 2000 rpm for 10 min at 4 °C, the lysate pellet was resuspended in 0.2 M acidic HCl solution overnight at 4 °C in rotation, for histone precipitation. Afterward, total protein and histone protein extracts were quantified by colorimetric detection using PierceTM BCA Protein Assay kit, according to the manufacturer’s instructions. Western blot was performed as described elsewhere^33^ to assess relative protein expression levels between different conditions. Detection was carried out using a Chemidoc system, after Clarity WB ECL chemiluminescent substrate addition (BioRad, Berkeley, California). Relative protein expression quantification was performed using ImageJ, version 1.53 (National Institutes of Health). β-actin and H3 were used as loading controls for total and histone protein extracts, respectively. At least three independent biological replicates were used in each assay. All original and uncropped films of Western blots from the main manuscript are presented in Supplementary Fig. S7. Antibody dilutions were optimized as detailed in Supplementary Table S2.

### Immunofluorescence microscopy

γ-H2AX foci, KDM6B and p53 were assessed through immunofluorescence staining. Fluorescent secondary antibodies—goat anti-rabbit immunoglobulin G (IgG) Alexa Fluor^TM^ 488 (A11008, Invitrogen, Thermofisher Scientific, USA), or goat anti-mouse IgG Alexa Fluor^TM^ 594 (A11032, Invitrogen, Thermofisher Scientific, USA) —were incubated for 1 h at room temperature in the dark with a final dilution of 1:1000 in 5% bovine serum albumin (BSA)/ PBS 1×. Experimental procedures were performed as previously described (5). All immunofluorescent images were taken in an Olympus microscope model IX51 (Tokyo, Japan). 4’6-diamidino-2-phenylindole (DAPI) (AR1176, BOSTER Biological Technologies, China) was used as a nuclear control dye for cell staining. All antibody dilutions were optimized as detailed in Supplementary Table S2.

### Alkaline comet assay (single-cell gel electrophoresis)

Briefly, 1 × 10^5^ 22Rv1-P and -RR cells were seeded in small culture flasks and, in the next day, treated with 10 μM of GSK-J4, followed by 2.5 Gy IR delivery after 24 h, when applicable. Then, at the end of the experiment, alkaline single-cell gel electrophoresis was performed to evaluate the percentage of DNA fragmentation as described elsewhere^33^. DAPI was used as a dye for nuclear DNA fluorescent staining. Representative images were taken in an Olympus microscope, model IX51 (Tokyo, Japan) and further analyzed with OpenComet Plugin for ImageJ version 1.53 (National Institutes of Health). At least 100 cells/ comets were considered per group for the analysis. Percentage DNA fragmentation data was summarized in tail moment measurements (tail length × % of DNA in the tail).

### ChIP (qPCR) and co-immunoprecipitation (Co-IP) for PCa cell lines

22Rv1-P and -RR cells were seeded in culture flasks at 1 × 10^7^ concentration. Chromatin immunoprecipitation (ChIP) protocol is detailed in a previous publication^33^. All primary antibodies were used at 1:100 in ChIP dilution buffer. Anti-RNA polymerase II and anti-mouse IgG antibodies were employed as internal positive and negative controls, respectively, for the immunoprecipitation (IP) reaction. For genomic DNA purification, Qiaquick gel extraction kit #28706 (Qiagen, Germany) was used according to the manufacturer’s instructions. Then, qRT-PCR was performed in 96-well plates in an ABI 7500 Real-Time PCR detection system (Applied Biosystems, Perkin Elmer, CA, USA). Each IP was run in triplicates. Primer sequences and melting temperatures are detailed in Supplementary Table S3. ChIP-qPCR data analysis was performed according to the percent Input method [100*2^ (Ct input-6.644-Ct (IP)].

Furthermore, KDM6B was immunoprecipitated with p53 in 22Rv1-P and -RR cell extracts. Briefly, the cells were harvested and resuspended in RIPA lysis buffer (Santa Cruz Biotechnology, USA), supplemented with 10% of protease inhibitor cocktail (PIC), for 15 min on ice, followed by sonication (2 × 10 s). Then, whole protein extract immunoprecipitation (IP) was carried out using Magn ChIP protein A + G beads (EMD Millipore, USA) and anti-KDM6B antibody (1:100), overnight at 4 °C on rotation. Anti-rabbit IgG antibody was used as the negative control for the IP reaction. The same amount of cell lysates was kept on ice for the input. In the next day, three wash steps (PBS 1× plus 0.5 M EDTA and PIC 10%) were performed for 5 min each, on ice. Then, pre-cleaned proteins with the beads were eluted with 20 μL of distilled water and 5 μL of loading buffer and incubated at 50 °C for 10 min. Lastly, the supernatant was used to perform SDS-PAGE Western blot, as previously described.

### RNA extraction, cDNA synthesis, and real-time quantitative polymerase chain reaction (RT-qPCR)

PCa cells were seeded in culture flasks at a concentration of 3 × 10^5^ cells and, at appropriated time-points, RNA extraction was performed. Total RNA extracts were obtained using Trizol reagent method. Then, starting from 1000 ng of RNA extracts, reverse transcription of cDNA was accomplished using Revert Aid RT Kit (Thermo Scientific Inc.) according to the manufacturer’s instructions. Afterward, qRT-PCR was performed in 96-well plates in an ABI 7500 Real-Time PCR detection system (Applied Biosystems, Perkin Elmer, CA, USA). Primer sequences and melting temperatures are described in Supplementary Table S3. RNA pool homemade containing different human cell lines was used as a positive control. A standard curve (serial dilutions 1:5) was included in each plate, in duplicates. Computed standard curve efficiency was validated when in a range of 90–100%. Absolute quantified values were achieved by standard curve interpolation. Data are represented as a relative quantification by the ratio between gene/*GUSB* (housekeeping) average multiplied by 1000, for easier tabulation. For each gene target, at least three independent biological replicates were used, and all samples were run in technical triplicates.

### siRNA transfection for *KDM6B* gene silencing

Briefly, three different siRNA oligos for *KDM6B* gene targeted CDS and 3’UTR regions (hs.Ri.KDM6B.13.1, hs.Ri.KDM6B.13.2 and hs.Ri.KDM6B.13.3), as well as one negative control siRNA sequence (DsiRNA, 1nmol), were purchased from Integrated DNA technologies (IDT) company, Coralville, IA, USA. Moreover, HPRT-S1 DS Positive Duplex and TYETM 563 DS Transfection were used as internal transfection controls, from the same manufacturer. Silencing of *KDM6B* gene expression by in vitro siRNA transfection was performed with Lipofectamine® 3000 (Invitrogen, USA), according to the manufacturer’s instructions. Gene silencing was confirmed for every experiment after 48 h of siRNA transfection.

### RT^2^ profiler PCR array

Human DNA damage signaling pathway array was used to determine transcriptional differences in DNA damage-related genes. RNA extracts from 22Rv1-P and 22Rv1-RR cell lines were used for this array. Briefly, 400 ng of RNA was collected to perform cDNA synthesis by RT^2^ First Strand kit (Qiagen, Germany), according to the manufacturer’s instructions. Then, RT^2^ Profiler PCR array was performed using Xpert Fast SYBER Mastermix Blue (GRISP) in QuantStudioTM Flex Real-Time PCR equipment (ThermoFisher Scientific). All samples passed in the genomic DNA contamination test, as well as in reverse transcription efficiency control. *HPRT1* was used as a housekeeping gene for normalization. Fold regulation values were calculated using 2^-ΔCt method for 22rv1-RR group compared to the control group (22Rv1-P).

### Immunohistochemistry (IHC) in FFPE tissue samples

Immunoexpression of p53, KDM6B, and H3K27me3 was evaluated in PCa FFPE tissue samples. IHC protocol was performed using NovolinkTM Max Polimer Detection System (Leica Biosystems). Citrate buffer 1x pH 8.0 was used for antigen retrieval during 20 min in a microwave for KDM6B and H3K27me3 antibodies, and in a water bath at 95 °C for p53 antibody. Antibody concentration details and positive control tissues are listed in Supplementary Table S2. Immunoexpression analysis was performed using a semi-quantitative method. Briefly, a dedicated Uropathologist revised all the slides and classified the staining according to the extension (% of positive cells: 0; 1–25; 25–50; 50–75; 75–100) and intensity score (0—null; 1—weak; 2—moderate; 3— strong). IHC score is a combination of both extension and intensity scores (E + I). Then, a cut-off for each protein was determined according to the distribution of the IHC score values, and for the final analysis samples were classified into high- or low-expressing cases.

### Cell viability assays

Resazurin dye solution (Canvax Biotech, Córdoba, Spain) was used to evaluate metabolically active cells as an indicator of cell viability. For this assay, 1 × 10^4^ cells were seeded in 96-well plates. Then, before any treatment or defined timepoint, OD values were obtained for each well representing 0 h timepoint for normalization. After 3 h of incubation at 37 °C, the solution containing resazurin in a proportion 1:10 with culture medium was transferred to a black 96-well plate, and measured using a microplate reader (Fluostar Omega, BMG Labtech, Germany) at 560 nm of wavelength. The viability of *TP53*-KD clones was determined in comparison with negative control samples until 48 h of cell replication. At least three independent biological replicates were used for this assay, each with experimental triplicates.

### Apoptosis and cell cycle assay by flow cytometry

Briefly, for both apoptosis and BrdU cell proliferation/ cell cycle assays, 7 × 10^4^ cells were seeded in six-well plates 24 h before GSK-J4 treatment and 48 h before 2.5 Gy of irradiation, when applicable. Then, 24 h after IR, cells were harvested and washed with cell staining buffer (Biolegend, Dedham, MA, USA).

To determine the percentage of apoptotic/necrotic cells, FITC Annexin V Apoptosis Detection Kit (Biolegend, Dedham, MA, USA) was used according to the manufacturer’s instructions. FITC Annexin V and 7-AAD were added in Annexin V Binding buffer for 15 min at room temperature. To assess cell proliferation/ cell cycle, Phase-Flow^TM^ FITC BrdU kit (Biolegend, San Diego, CA, USA) was used according to the manufacturer’s protocol. BrdU was incorporated in the cells for 1h30 min at 37 °C. Then, after fixation and permeabilization, cells were stained with FITC anti-BrdU antibody, and 7ADD was added. Cells were acquired using a FACS Canto^TM^ II Cell Analyzer (BD Bioscience, Franklin Lakes, NJ, USA), and analyzed using FlowJo^TM^ software (BD Biosciences, Franklin Lakes, NJ, USA). Cells were pre-gated as single cells. At least three independent biological replicates were used in each assay.

### In vivo tumor growth assessment using chicken chorioallantoic membrane (CAM) assay

To induce 3D microtumor formation, 2 × 10^6^ of 22Rv1-P and RR PCa cells were injected into chicken chorioallantoic membrane (CAM) at the 10th day of embryo development. Thus, freshly fertilized eggs were incubated at 37 °C in a humid environment for a maximum of 17 days of embryo growth. 22RV1-P and -RR cells were consecutively treated with 10 μM of GSK-J4 and/or 2.5 Gy of irradiation, when applicable. Respective controls with either DMSO or PBS 1× in RPMI were added to the remaining groups. A total of 80 eggs were randomly divided into ten eggs per group. Representative images of microtumors among different groups were taken by a stereomicroscope Olympus S2X16 using a digital camera Olympus SC180. Tumor area was measured using ImageJ tools, version 1.53 (National Institutes of Health). 22Rv1-P and -RR microtumors were irradiated using the same previously established conditions^33^. For this work, eggs were irradiated using microSelectronv3 Iridio-192 brachytherapy (192-Ir-mHDR-v2r) with Air kerma rate constant of 4.082 cGy cm^2^/h/mCi and 2.5 Gy per egg/pulse delivered until 9.5 mm distance from the top of eggshell (where the microtumor is located). Then, the absorbed dose was predefined to be consecutively decreasing over larger distances for the chicken embryo’s protection^33^. The physician irradiation plan is represented in Supplementary Fig. S8. At the end of each experiment, all the microtumors were dissected from the egg and included in a paraffin block for subsequent performance of tissue sections. Then, immunostaining was carried out as previously described for the patient-derived specimens and evaluated using a quantitative method from GenASIS software (Applied Spectral Imaging, ASI), considering the percentage of positive cells.

### Statistical analysis

Radiobiological cell survival curves were analyzed using linear-quadratic (LQ) model (S = e ^– (αD + βD2)^). When applicable, nonparametric tests including the Mann–Whitney *U* test and the Kruskal–Wallis test, with Dunn’s correction, were used to compare only between two groups or between three or more groups, respectively. For all the analyses, *P* values inferior to 0.05 were considered statistically significant (*P < 0.05; **P < 0.01; ***P < 0.001; ****P < 0.0001; ns, not significant). All data were analyzed using GraphPad Prism software version 9.1.1.

### Supplementary information

Supplementary Materials for Epigenetic regulation of TP53 is involved in prostate cancer radioresistance and DNA damage response signaling

## Data Availability

The authors confirm that the data supporting the findings of this study are available within the article and in the Supplementary material file. Raw data that support the findings of this study are available from the corresponding author, upon reasonable request.
