# Supplementary material for: Epigenetic regulation of TP53 is involved in prostate cancer radioresistance and DNA damage response signaling
Source: Signal Transduct Target Ther. 2023 Oct 16;8:395. doi: 10.1038/s41392-023-01639-6 (PMC10577134; doi:10.1038/s41392-023-01639-6)
Supplement: Supplementary file 1 — Supplementary Materials for Epigenetic regulation of TP53 is involved in prostate cancer radioresistance and DNA damage response signaling [file 41392_2023_1639_MOESM1_ESM.docx]

Supplementary Materials for

Epigenetic regulation of TP53 is involved in prostate cancer radioresistance and DNA damage response signaling

Catarina Macedo-Silva; Vera Miranda-Gonçalves; Nuno Tiago Tavares; Daniela Barros-Silva; Joana Lencart; João Lobo; Ângelo Oliveira; Margareta P. Correia; Lucia Altucci; Carmen Jerónimo

Correspondence to: carmenjeronimo@ipoporto.min-saude.pt / [cljeronimo@icbas.up.pt](mailto:cljeronimo@icbas.up.pt)

**This PDF file includes:**

Supplementary Figures. S1 to S8

Supplementary Figures captions

Tables S1 to S3 and respective captions

References for Supplementary table S1

.


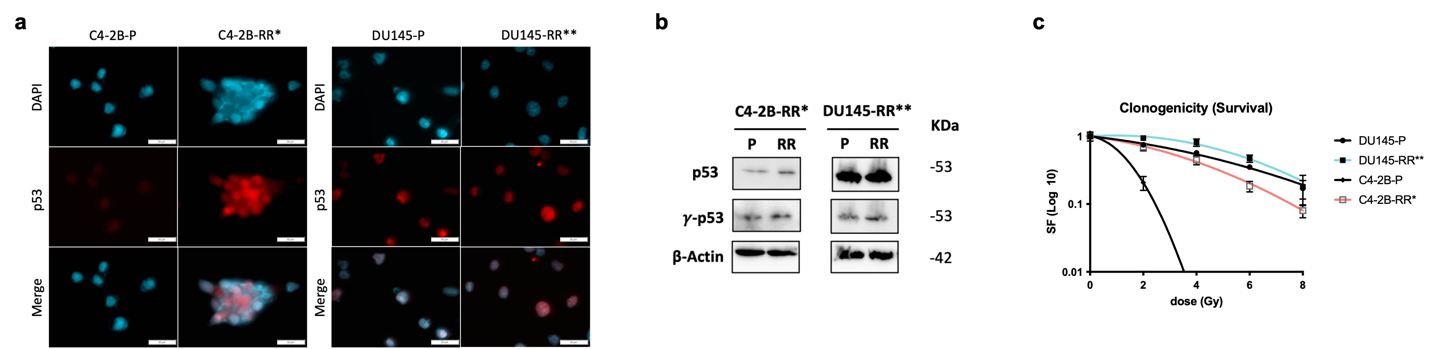
Figure. S1.

**Supplementary fig. S1. *In vitro* generation of radioresistant (RR) cell** **line** **subpopulations**s **and characterization of IR-induced p53 expression.** a) Immunofluorescence staining p53 (merged with DAPI) in C4-2B and DU145 cells, both parental (P) and radioresistant (RR) fractions. Images were taken using Olympus IX51 microscope at 200x magnification (scale bar 50 μm). b) Total protein levels of p53 (53kDa) and γ-p53 (53kDa) in C4-2B and DU145 cells, both parental (P) and radioresistant (RR) fractions. β-actin (42kDa) was used as loading control. Images were taken by Chemidoc detection system (Biorad, Berkeley, California). c) Cell survival fraction of C4-2B and DU145, parental (P) and radioresistant (RR) cell fraction, represented through linear-quadratic model (LQ = (S=e ^– (αD + βD2)^)). RR*, cells were submitted to 10 fractions of 2.5Gy/fraction. RR**, cells were submitted to 20 fractions of 2.5Gy/fraction.

Figure. S2.


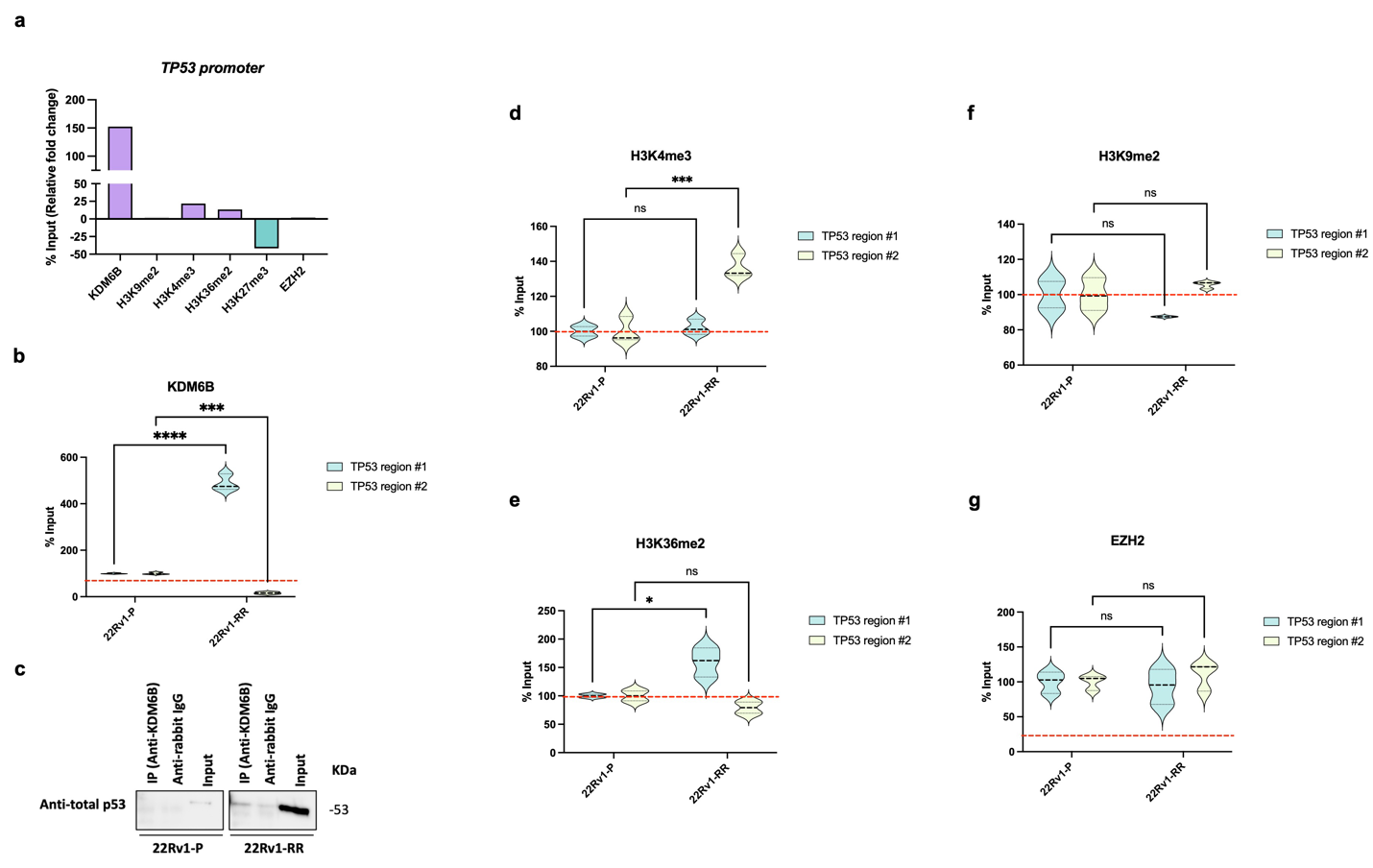


**Supplementary fig. S2. Epigenetic regulation of *TP53* gene promoter.** a) Relative fold change % input values of 22Rv1-RR vs 22Rv1-P for KDM6B, H3K9me2, H3K4me3, H3K36me2, H3K27me3 and EZH2 binding at *TP53* gene promoter considering the bulk of both regions. b) KDM6B immunoprecipitation % Input (ChIP-qPCR) at *TP53* gene promoter in regions #1 and #2 (180bp and 360bp upper TSS, respectively) in 22Rv1-P and RR cells. c) Co-Immunoprecipitation (Co-IP) blots for KDM6B and p53 in 22Rv1-P and -RR cells. Anti-rabbit IgG was used as negative control. Input was used as loading protein control for the p53 immunoexpression (53kDa). d-g) H3K4me3, H3K9me2, H3K36me2 and EZH2 immunoprecipitation % Input (ChIP-qPCR) at *TP53* gene promoter in regions #1 and #2 (180bp and 360bp upper TSS, respectively) in 22Rv1-P and RR cells. The violin plots depict mean ± SD of at least three independent replicates. ns, non-significant; * p value <0.05; *** p value <0.001; **** p value <0.0001. H) Co-Immunoprecipitation (Co-IP) blots for KDM6B and p53 in 22Rv1-P and -RR cells. Anti-rabbit IgG was used as negative control. Input was used as loading protein control for the p53 immunoexpression (53kDa).

Figure. S3.


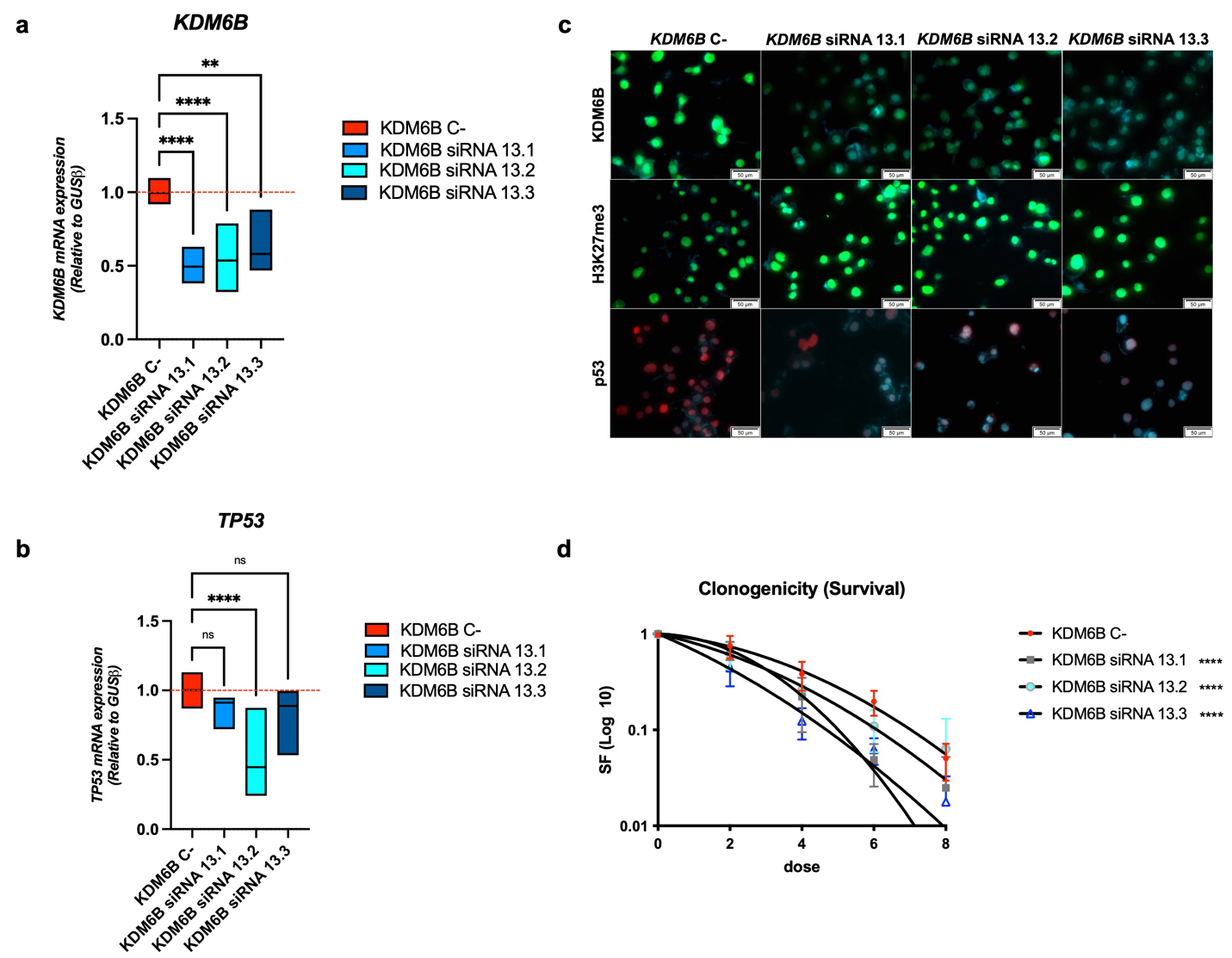


**Supplementary fig. S3. *KDM6B* silencing and its impact on *TP53* expression and on radiotherapy response.** a-b) Relative mRNA expression levels of *KDM6B* and *TP53* genes for 22Rv1-RR negative control (C-) and KDM6B knocking down siRNAs. Results are presented as mean ± SD of at least 3 independent experiments. *GUSB* was used as reference gene for normalization. c) Immunofluorescent staining of KDM6B (green), H3K27me3 (green) and p53 (red) merged with DAPI for nucleus location in 22Rv1-RR negative control (C-) and *KDM6B* knocking down siRNAs. Images were taken using Olympus IX51 microscope at 200x magnification (scale bar 50 μm). d) Cell survival fraction of 22Rv1-RR negative control (C-) and KDM6B knockdown cells, represented through linear-quadratic model (LQ = (S=e ^– (αD + βD2)^)).

Figure. S4.


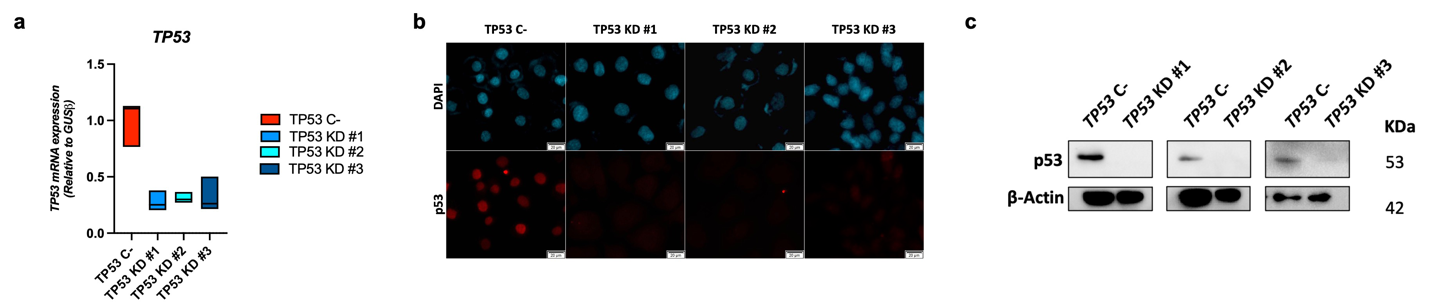


**Supplementary fig. S4. Generation of stable *TP53*-Knockdown cell lines using CRISPR/Cas9 technology.** a) Relative mRNA expression levels of *TP53* gene for 22Rv1-RR negative control (C-) and TP53-KD clones #1 to #3. Results are presented as mean ± SD of at least 3 independent experiments. *GUSB* was used as reference gene for normalization. b) Nuclear immunofluorescent staining of p53 (red) and DAPI (blue) for 22Rv1-RR negative control (C-) and TP53-KD clone #1 to #3. Images were taken using Olympus IX51 microscope at 400x magnification (scale bar 20 μm). c) Total protein levels of p53 (53kDa) of 22Rv1-RR negative control (C-) and TP53-KD clone #1 to #3 cells. β-actin (42kDa) was used as loading control. Images were taken by Chemidoc detection system (Biorad, Berkeley, California).

Figure. S5.


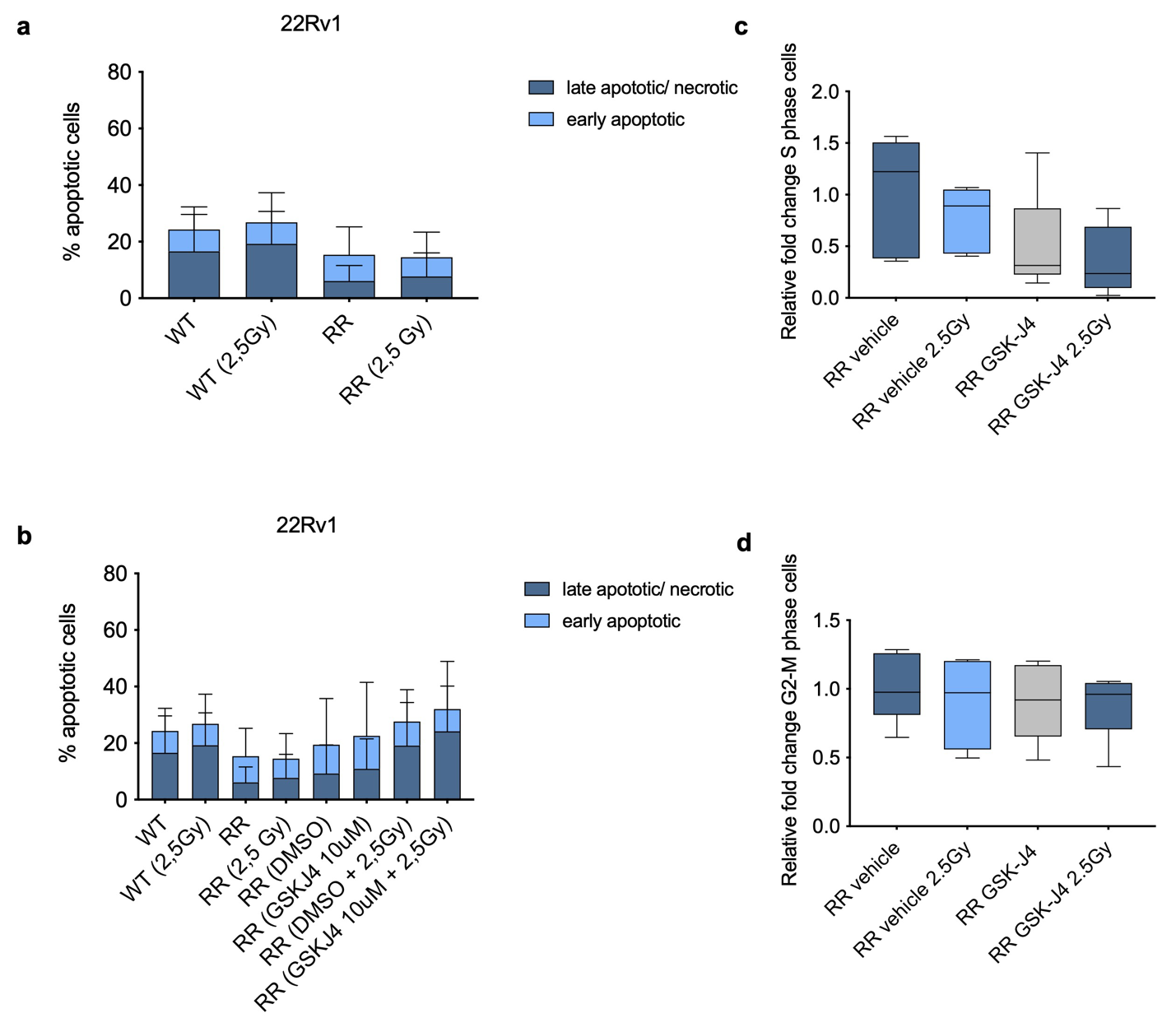


**Supplementary fig. S5. Cell cycle and apoptotic patterns in 22Rv1-P and -RR cell lines with/without GSK-J4 and/or 2.5Gy.** Data quantification by flow cytometry a, b) for early and late apoptotic % cells in 22Rv1-P and RR cells with/without GSK-J4 treatment (DMSO was used as vehicle control for GSK-J4 compound); c) Relative fold-change of % of 22Rv1-RR cells in c) S phase and d) G2-M phase with/without GSK-J4 and with/without 2.5Gy.

Figure. S6.


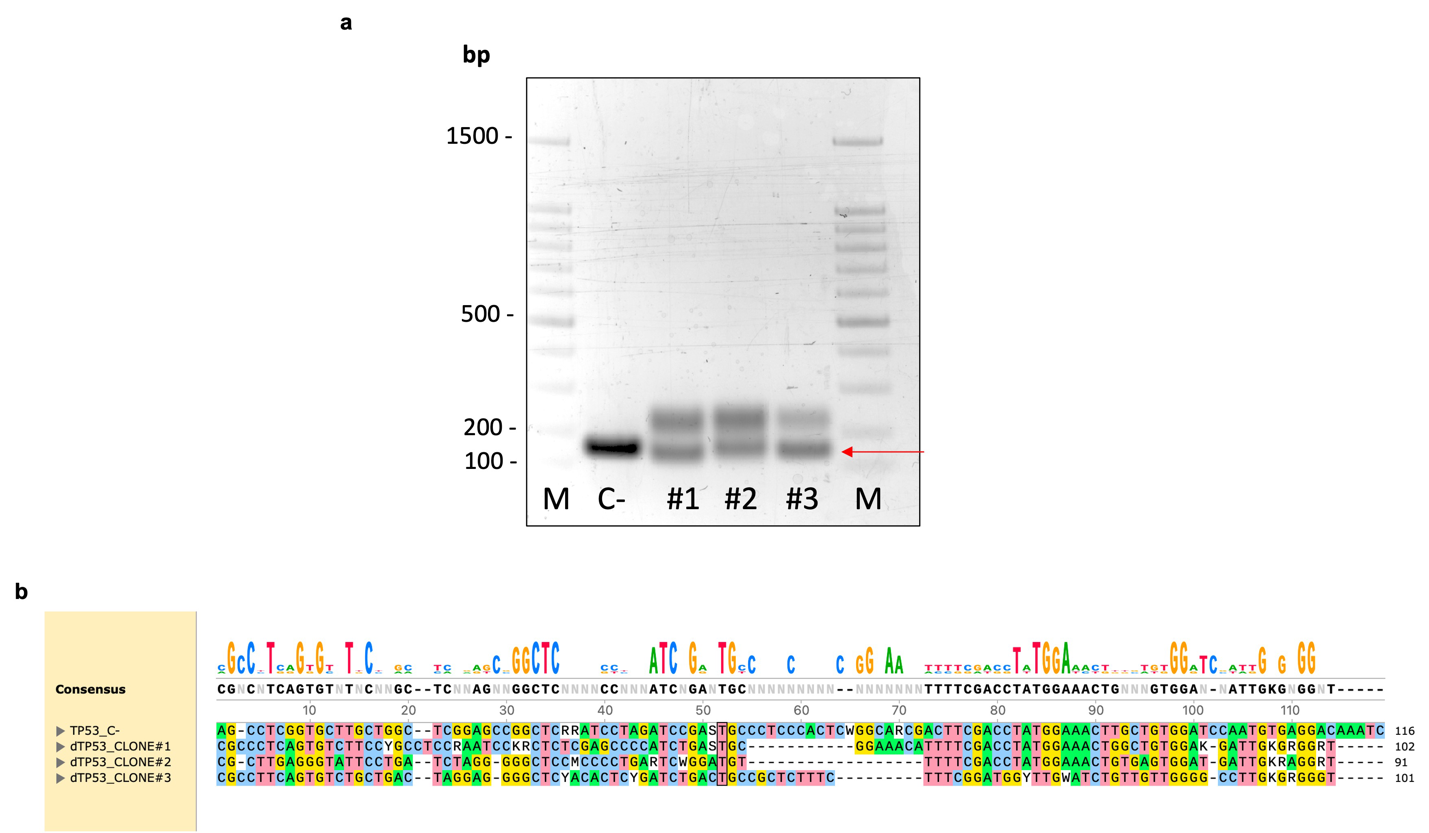


Supplementary fig. S6. Genomic DNA sequencing to confirm TP53 deletion by CRISPR/Cas9. a) Electrophoresis gel with PCR products obtained from conventional PCR using MyTaq HS Red polymerase to evaluate TP53 genomic sequence. Sample #1 is the negative control (wild-type 22Rv1-RR cells) with complete WT sequence of TP53. The followed three samples represent TP53-KD clones #1 to #3 with heterozygous deletion of TP53 b) Alignment sequences for TP53 negative control, C- and TP53-KD clones #1, #2 and #3. Alignment adapted from SnapGene software, version 6.2.2. Abbreviations: bp, base pairs; M, marker.

Figure. S7.


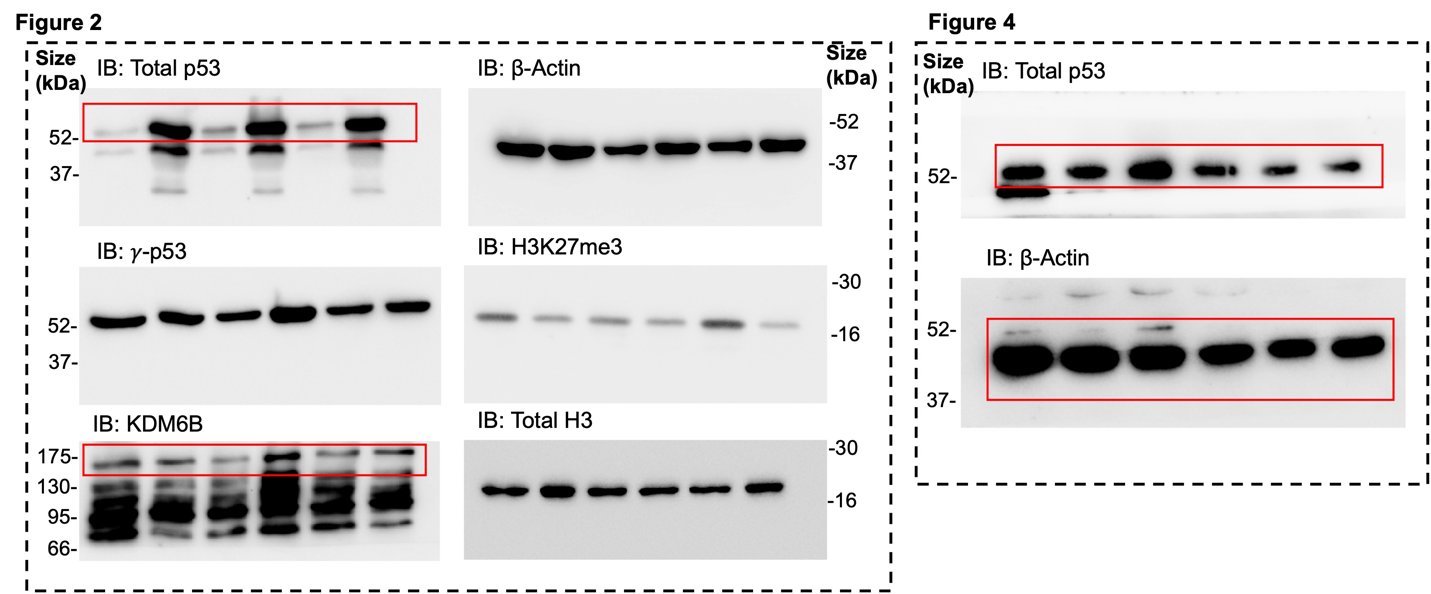


**Supplementary fig. S7. Original and uncropped films of Western blots of the main manuscript**. Each immunoblotting panel (IB) represents three independent paired biological replicates (Parental and radioresistant from figure 2 and Vehicle and GSK-J4 from figure 4). For the main figures were selected the best representative pairs.

Figure. S8.


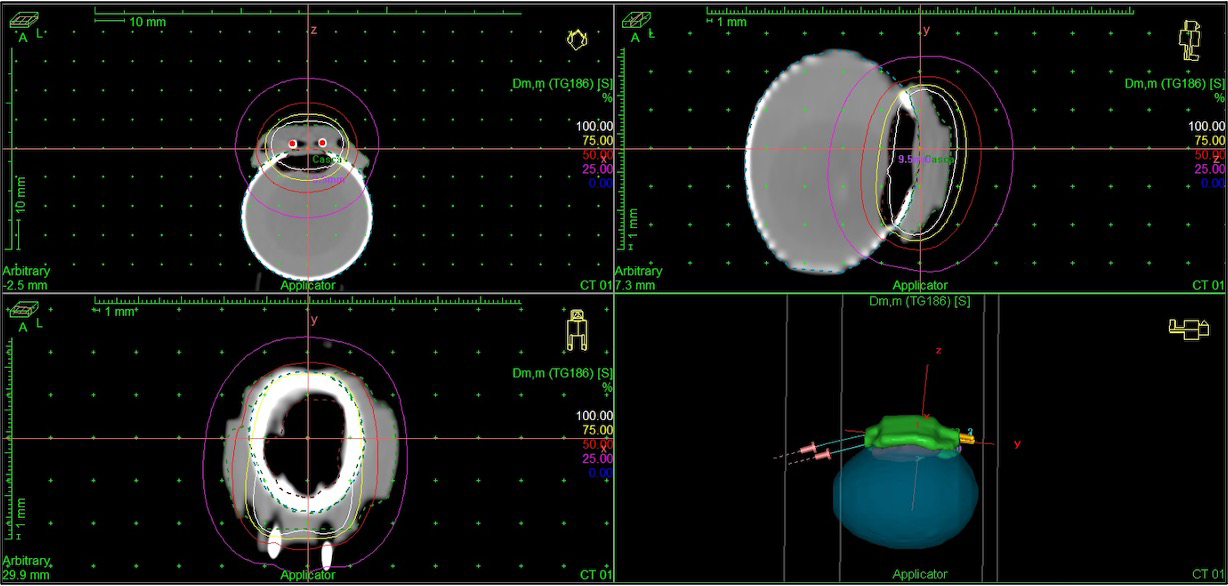


**Supplementary fig. S8. *In vivo* CAM assay microtumors irradiation.** 3D conformation treatment planning of CAM assay with 2.5Gy irradiation in single-dose in microSelectronv3 Iridio-192 brachytherapy (192-Ir-mHDR-v2r). The four quadrants refer to the different treatment plan angles and incident areas of ionizing radiation that involves the tumor area in the chicken embryo chorioallantoic membrane. This specific zone was irradiated in a range of approximately 100% (white) of the planned dose. Further, in depth, there is a decrease in the absorbed dose to 75% (yellow), 50% (red) and 25% (purple), until no dose reached. In this way, the chicken embryo receives a minimal dose of radiation since it is located at the bottom of the egg, due to gravity.

Table S1. Genes that satisfy the conditions of fold-change two times greater and p value lower than 0.05 based on the Human DNA Damage Signaling Pathway RT^2^ Profiler PCR array.

| **Gene** | **Fold regulation** | ***p value*** | **Main Function** | **Ref.** |
| --- | --- | --- | --- | --- |
| *ABL1* | 3.51 | 0.002 | Tyrosine-protein kinase (cell growth and several other functions) | [1] |
| *CSNK2A2* | 2.31 | 0.003 | Protein kinase (DNA damage) | [2] |
| *ERCC1* | 3.11 | 0.010 | DNA repair nuclease (NER pathway) | [3] |
| *MSH2* | 2.19 | 0.004 | DNA damage mismatch repair pathway | [4] |
| *RAD1* | 2.45 | 0.004 | Checkpoint DNA exonuclease | [5] |
| *RBBP8* | 2.39 | 0.008 | DNA endonuclease | [6] |
| *SUMO1* | 2.22 | 0.030 | Ubiquitin protein; post translational modification | [7] |
| *TP53* | 5.83 | 0.001 | Response to cellular stress | [8] |
| *BBC3* | -4.10 | 0.023 | Pro-apoptotic protein | [9] |
| *CDC25A* | -2.37 | <0.001 | Cell cycle checkpoint | [10] |
| *CRY1* | -2.55 | 0.003 | Circadian factor control; DNA repair regulator (G2/M transition) | [11] |
| *LIG1* | -2.96 | 0.001 | DNA replication; recombination; BER | [12] |
| *MDC1* | -2.10 | 0.012 | Mediator of DNA damage checkpoint | [13] |
| *MLH1* | -5.17 | <0.0001 | DNA damage mismatch repair pathway | [4] |
| *PARP1* | -2.02 | 0.004 | Broad cellular functions | [14] |
| *RAD18* | -2.03 | <0.0001 | Post replication repair | [15] |

References for Supplementary table S1:

1 Wang, J. & Pendergast, A. M. The Emerging Role of ABL Kinases in Solid Tumors. Trends Cancer **1**, 110-123 (2015).

2 Rabalski, A. J., Gyenis, L. & Litchfield, D. W. Molecular Pathways: Emergence of Protein Kinase CK2 (CSNK2) as a Potential Target to Inhibit Survival and DNA Damage Response and Repair Pathways in Cancer Cells. Clin Cancer Res **22**, 2840-2847 (2016).

3 Jacobsen, F. et al. Increased ERCC1 expression is linked to chromosomal aberrations and adverse tumor biology in prostate cancer. BMC Cancer **17**, 504 (2017).

4 Dominguez-Valentin, M. et al. Frequent mismatch-repair defects link prostate cancer to Lynch syndrome. BMC Urol **16,** 15 (2016).

5 Cabañas Morafraile, E. et al. Genomic Correlates of DNA Damage in Breast Cancer Subtypes. Cancers (Basel) **13** (2021).

6 Zarrizi, R. et al. Germline RBBP8 variants associated with early-onset breast cancer compromise replication fork stability. J Clin Invest **130**, 4069-4080 (2020).

7 Burdelski, C. et al. The prognostic value of SUMO1/Sentrin specific peptidase 1 (SENP1) in prostate cancer is limited to ERG-fusion positive tumors lacking PTEN deletion. BMC Cancer **15,** 538 (2015).

8 Teroerde, M. et al. in Prostate Cancer (eds S. R. J. Bott & K. L. Ng) (Exon Publications

Copyright: The Authors., 2021).

9 Shan, Z. et al. PUMA decreases the growth of prostate cancer PC-3 cells independent of p53. Oncol Lett **13,** 1885-1890 (2017).

10 Nemoto, K. G2/M accumulation in prostate cancer cell line PC-3 is induced by Cdc25 inhibitor 7-chloro-6-(2-morpholin-4-ylethylamino) quinoline-5, 8-dione (DA 3003-2). Exp Ther Med **1**, 647-650 (2010).

11 Shafi, A. A. et al. The circadian cryptochrome, CRY1, is a pro-tumorigenic factor that rhythmically modulates DNA repair. Nat Commun **12**, 401 (2021).

12 Yadav, S. et al. Somatic mutations in the DNA repairome in prostate cancers in African Americans and Caucasians. Oncogene **39**, 4299-4311 (2020).

13 Wang, C. et al. MDC1 functionally identified as an androgen receptor co-activator participates in suppression of prostate cancer. Nucleic Acids Res **43,** 4893-4908 (2015).

14 Deshmukh, D. & Qiu, Y. Role of PARP-1 in prostate cancer. Am J Clin Exp Urol **3**, 1-12 (2015).

15 Tripathi, K., Mani, C., Clark, D. W. & Palle, K. Rad18 is required for functional interactions between FANCD2, BRCA2, and Rad51 to repair DNA topoisomerase 1-poisons induced lesions and promote fork recovery. Oncotarget **7,** 12537-12553 (2016).

Table S2. Antibodies details for the different applications.

| **Antibody** | **Reference** | **IHC** | | | **WB dilution** | **IF dilution** |
| --- | --- | --- | --- | --- | --- | --- |
|  |  | **Dilution** | **Antigen retrieval buffer** | **Positive control tissue** |  |  |
| β-Actin | A1978, Sigma-Aldrich | - | - | - | 1:10000 | - |
| γ-H2AX | 2577s, cell signaling | - | - | - | - | 1:500 |
| H3 | abcam | - | - | - | 1:3000 | - |
| H3K27me3 | 07-449, Millipore | 1:1000 | Citrate 1x, pH8; Mw:20min | Normal testis | 1:500 | - |
| KDM6B/JMJD3 | ab38113, abcam | 1:1000 | Citrate 1x, pH8; Mw:20min | Colorectal cancer | 1:1000 | 1:250 |
| p53 | M700129-2 (DO-7) Lusopalex | 1:200 | Citrate 1x, pH8; WB:20min | Serous carcinoma | 1:500 | 1:250 |
| γ-p53 | 9286T (Ser15), cell signaling | - | - | - | 1:1000 | - |

Mw, microwave; WB, Western blot; IF, immunofluorescence; IHC, immunohistochemistry.

Table S3. RT-qPCR and ChIP-qPCR (Fw and Rv) primers details.

| **Application** | **Gene name** | **Region** | **Distance from TSS (bp)** | **Fw sequence (5’ ->3’)** | **Rv sequence (5´->3’)** | **Annealing temperature (Cº)** |
| --- | --- | --- | --- | --- | --- | --- |
| ChIP-qPCR | *TP53* | #1 | 180 | CCTCCCATGTGCTCAAGACT | CAGGAGCTTACCCAATCCAG | 60 |
|  | *TP53* | #2 | 360 | CAGAATTTTCCACCCCAAAA | TGGCACAAAGCTGGACAGT | 60 |
| RT-qPCR | *TP53* | - | - | CGCTTCGAGATGTTCCGAGA | CTTCAGGTGGCTGGAGTGAG | 60 |
|  | *GUSB* | - | - | CACTGAAGAGTACCAGAAAAGTC | TCTCTGCCGAGTGAAGATCC | 60 |

Bp, base pairs; TSS, transcription starting site.
